# CoCoss-Trial: Concurrent Comparison of Self-Sampling Devices for HPV-Detection

**DOI:** 10.3390/ijerph181910388

**Published:** 2021-10-02

**Authors:** Faruk Cem Ertik, Johanna Kampers, Fabienne Hülse, Claudia Stolte, Gerd Böhmer, Peter Hillemanns, Matthias Jentschke

**Affiliations:** 1 Department of Gynaecology and Obstetrics, Hannover Medical School, 30625 Hannover, Germany; Faruk.C.Ertik@stud.mh-hannover.de (F.C.E.); Kampers.Johanna@mh-hannover.de (J.K.); Huelse.Fabienne@mh-hannover.de (F.H.); Hillemanns.Peter@mh-hannover.de (P.H.); 2 IZD Hannover, 30159 Hannover, Germany; Stolte@izd-hannover.de (C.S.); Boehmer@izd-hannover.de (G.B.)

**Keywords:** HPV-testing, self-sampling, cervical cancer screening, hr-HPV

## Abstract

High-risk human papillomavirus (hr-HPV) infection of the cervicovaginal tract is known to be the major cause of cervical cancer. Similar to various other countries, Germany introduced an organized combined screening including cytology and HPV testing in 2020. The participation rate was around 70% in the past. Self-testing for hr-HPV infections could be an option to increase the participation rate. Two dry vaginal self-sampling devices and a device for the self-collection of first-void urine were evaluated in combination with a PCR-based hr-HPV test regarding their clinical performance (sensitivity for high-grade cervical intraepithelial neoplasia, CIN 2+). A cervical smear taken by a clinician during colposcopy was used as reference. This open prospective multicenter trial recruited patients referred to the two participating colposcopy clinics (Hannover Medical School and IZD Hannover, Germany) with abnormal results from cervical cancer screening from 05/2020 to 11/2020. All patients received three CE-certified self-sampling devices (FLOQSwabs, COPAN, Italy; Evalyn Brush, Rovers Medical Devices, the Netherlands; Colli-Pee FV-5000, Novosanis, Wijnegem, Belgium) with instructions to read and apply at home in a pre-specified alternating order without medical assistance. HPV testing was performed after adequate preservation and DNA extraction. Histological results from colposcopy or cervical excisional surgery after self-sampling were used as the gold-standard. The data of 65 patients were analyzed. All invasive cancer cases and over 90% of the CIN 3 lesions were found to be hr-HPV positive with all three self-collection devices. All devices were considered easy to use without any difficulties following the written instructions. Hr-HPV testing of self-collected first-void urine and dry vaginal self-samples showed a high sensitivity for CIN 3+ comparable to that of a clinician-taken smear. Self-sampling was well accepted as it is convenient and easy to use.

## 1. Introduction

High-risk human papillomavirus (hr-HPV) infection of the cervicovaginal tract is known to be the major cause of cervical cancer [1]. The new German screening program introduced in 2020 is based on the knowledge of the superior efficacy of a cervical HPV-smear to reduce the incidence of cervical cancer in comparison to cervical cytology [2,3,4]. Similar to various other countries, Germany introduced an organized combined-testing including cytology and HPV scrape. From age 20 to 35, women continue to have yearly cytological testing, whereas women older than 35 receive a combined cytological and HPV testing every three years. Screening intervals and testing methods were chosen to minimize an overestimated prevalence of HPV, especially in women <30 years [5]. Participation rate is in need of improvement. In data from 2004 to 2009, among all patients with invasive cancer, 60% have not been screened for at least five years [6]. In a survey among 20,000 women in Germany, 74% stated to have participated in cervical carcinoma screening at least once in three years [7]. Another survey, which analyzed the dataset of a statutory health insurance in Lower Saxony, Germany, with case numbers between 940,000 (2006) and 1,045,000 (2011), showed a decrease in participation rate with higher age or lower socioeconomic status [8].

Cervicovaginal self-sampling could be an option to increase participation rate. Not only is the patients’ opinion towards this method positive [9], but also the willingness to participate has been higher in patients not attending screening regularly compared to a regular call for physician taken cytology [10]. HPV testing on self-collected and physician-collected cervical specimens with clinically validated PCR-based tests shows an overall similar sensitivity for CIN 3 on both sampling methods [11,12,13]. A different option is to self-collect first-void urine for HPV-testing. Several studies in which participants with abnormal cytology or self-reported HPV infection used urine sampling report an acceptable sensitivity of detection of high-grade cervical lesions and patients’ acceptability [14,15].

The EVAH study showed that physician-taken smears, brush-based self-sampling and first-void urine are equally sensitive to detect CIN 2+ using two different hrHPV tests (the highly sensitive SPF10 LiPA 25, version 1 assay or the clinically validated GP5+/6+ based luminex assay). None of the samples or assays missed CIN 3 [16].

Most published self-sampling studies have used the Evalyn Brush (Rovers Medical Devices, the Netherlands) and in several countries, such as the Netherlands, this device is already used for regular self-sampling of screening non-responders. Therefore, the Evalyn Brush can be regarded as an established self-sampling device for reference.

This study compared a dry vaginal self-sampling device (FLOQSwabs, COPAN, Brescia, Italy) and a device for the self-collection of first-void urine (Colli-Pee FV-5000, Novosanis, Wijnegem, Belgium) with the Evalyn Brush in combination with a PCR-based high-risk HPV Test to evaluate the clinical performance (sensitivity for high-grade CIN) of these devices towards the reference device. A cervical smear taken by a clinician during colposcopy was a separate reference for HPV testing.

## 2. Materials and Methods

This open prospective multicenter phase II trial recruited patients referred to the two participating colposcopy clinics (Hannover Medical School and IZD Hannover) with an abnormal cervical cytology from May 2020 to November 2020. The study was set up as a pilot study to compare three different self-sampling devices among a study population with a high risk of HPV infection and present cervical dysplasia in preparation for a larger population-based trial planned in the future. This trial is registered at the German register of clinical studies (“DRKS”, registration number: DRKS00019200). All patients gave written informed consent. Exclusion criteria were pregnancy or women with hysterectomy in the past. All patients received the three CE-certified self-sampling devices, as previously described, with instructions to read and apply at home in a pre-specified alternating order without medical assistance. The urine test should always be performed first, and after that the two swabs alternated.

The self-sampling was performed at home the day before a planned colposcopy or cervical excisional surgery. Patients were informed to avoid intimate cleaning before using the devices–especially the urine collection device. HPV testing was performed after adequate preservation and DNA extraction.

The Evalyn Brush has a soft and flexible collection head that can be retracted, and insertion indicator wings to ensure correct depth. Cell material is collected by rotating the brush five times then retracting the device and sealing it with a cap. It can then be sent by mail.

The FLOQSwab is an 8 cm long and few millimeters wide staff with short fiber strands at one end to absorb dislodged cells. The swab end has to be inserted as far as possible without using excessive force to collect cell material, then the device is removed and put into the transport test tube without further manipulation. The transport test tube can be sent by mail.

The Colli-Pee FV-5000 is a non-invasive self-sampling device with a funnel shaped collector tube. It is designed to collect 18–22 mL of first-void urine; then, excess urine runs past the sample tube. The sample tube can be disconnected from the device afterwards and closed with a cap. The DNA contained in the urine is stabilized at room temperature for a maximum of seven days by 7 mL of urine conservation medium (UCM) prefilled in the device by the manufacturer. The sample tube can also be sent by mail, the rest of the device can be disposed of.

Briefly, the Evalyn Brush and FLOQSwab samples were both transferred to 20 mL of Cytyc ThinPrep PreservCyt Solution (Hologic, Marlborough, MA, USA) from which 500 µL were used for DNA extraction and hr-HPV testing. For the first-void urine conserved in UCM, 500 µL was taken directly from the sample for DNA extraction and hr-HPV testing. Urine and dry-vaginal self-sampling devices were processed, as previously described, as soon as possible but all within seven days after collection. All self-collected samples were used without further preliminary analyses (DNA quality/integrity etc.), as earlier studies have shown excellent quality of dried samples even after a longer storage time [17].

A liquid-based cervical cytology smear was taken with a broom-like device (Hologic, Marlborough, MA, USA) and immediately suspended in 20 mL of ThinPrep medium. The physician-collected reference samples were used to prepare liquid-based cytology slides with the ThinPrep 2000 system (Hologic). From this material, both cytological and hrHPV-testing were performed and used for reference in our study. All samples were tested with the Abbott RealTime High Risk HPV Test (Abbott GmbH & Co. KG, Wiesbaden, Germany), an automated, qualitative multiplex PCR assay for the detection of 14 hr-HPV genotypes (16, 18, 31, 33, 35, 39, 45, 51, 52, 56, 58, 59, 66 and 68) and simultaneous differentiation of HPV 16 and HPV 18, as previously described [18]. An example of the amplifications curves can be found in the Supplemental Material.

Then colposcopy was performed using acetic acid and taking directed biopsies and/or endocervical curettage if indicated (routine cyto- and histopathological examination). In the case of unsuspicious colposcopy there was no biopsy taken. Biopsy specimen were stored in separate vials, fixed with 4% formalin and processed by the Department of Pathology of Hannover Medical School and IZD Hannover. Hematoxylin-eosin sections were prepared and classified by a member of the Department of Pathology. Histology was classified into low-grade, moderate and high-grade cervical intraepithelial neoplasia (CIN 1–3), adenocarcinoma in situ and invasive cancer [19]. The final diagnosis for each woman was defined according to the worst histological or cytological result.

The women were asked to fill out two short quantitative questionnaires to investigate acceptability of the three respective devices. The devices had to be rated according to the difficulty of use ranging from 1 (very difficult) to 10 (very easy), the comprehensibility of the instructions from 1 (very difficult) to 10 (very easy), the certainty of generating a valid sample from 1 (very uncertain) to 10 (very certain) and the comfortability of use from 1 (very uncomfortable) to 10 (very comfortable), respectively. Patients were also asked for their preferred screening method (including physician’s swab) and personal anamnesis.

Microsoft Excel 2014 (Microsoft Corp., Redmond, WA, USA) was used for data collection. Cohen’s kappa (K) was used to measure agreement of self- and physician-collected specimen regarding hrHPV positivity. Bias corrected 95% confidence intervals (CI) were estimated using Microsoft Excel 2014. The sample size was powered to detect substantial agreement (κ = 0.80) between self- and physician-collected specimen with a 5% a significance level. Results were considered significant if the *p*-value was lower than α = 0.05. The efficacy of hr-HPV testing for CIN 2+ detection was evaluated as sensitivity and specificity.

## 3. Results

### 3.1. Patient Characteristics

Seventy patients were recruited to the study. Five patients were excluded because of incomplete testing results (invalid questionnaire in one case and invalid or missing clinician-taken smear for reference in four cases), which led to a final number of 65 patients whose data was analyzed. The median age was 36 years (range, 24–76 years). Papanicolaou (PAP) smear results were I-IIa in 4, II-p in 0, III-g in 2, III-p in 3, IIID1 in 6, IIID2 in 24, IVa in 26 and V in 0 cases.

### 3.2. HPV Prevalence

Without consideration of the genotype, the hr-HPV detection was positive in 56 (86.2%) cases of physician swabs. In total, 56 (86.2%) of Evalyn Brush, 51 (78.5%) FLOQSwab and 48 (73.8%) Colli-Pee results were positive, respectively (Table 1). The histological results were 5 CIN 1, 28 CIN 2, 24 CIN 3, 1 adenocarcinoma in situ, 5 squamous cell carcinoma and 2 benign lesions, respectively. All invasive carcinomas and over 90% of the CIN 3 lesions were found to be hr-HPV positive with all self-collection devices as shown in Table 1.

Comparing the signal intensity for ß-globin (necessary number of real-time PCR cycles (CN) until the amplified DNA fragments reach the predefined cutoff values; this number inversely correlates with the amount of DNA in the sample) across all devices showed significantly higher concentration of DNA in Evalyn Brush samples (mean CN: 21.8; 95% CI: 21.4–22.3) than in FLOQSwab (mean CN: 23.1; 95% CI: 22.5–23.6) or Colli-Pee (mean CN: 23.7; 95% CI: 23.2–24.2) *p* < 0.001 and *p* < 0.001, respectively. There is no significant difference between FLOQSwab and Colli-Pee cycle numbers (*p* = 0.054).

### 3.3. Detection Rate of CIN 2+ Lesions

Sensitivity/Specificity of hr-HPV testing for CIN 2+ detection in clinician-taken smears was 89.7%/42.9%, in self-taken smear 89.7%/42.9% for the Evalyn-Brush, 82.8%/71.4% for the FLOQSwab and 77.6%/57.1% for the first-void urine as shown in Table 2. There were no significant differences in sensitivity or specificity for CIN 2+ detection between the self-smears nor between the clinician-taken smears and the device samples (see Table 3).

### 3.4. Agreement on HPV Detection

HPV detection concordance and the agreement between specimen types is shown in Table 4. The agreement between clinician-taken and self-samples was fair to moderate (K 0.29–0.48). The agreement between the self-samples was moderate to substantial (K 0.57–0.75).

### 3.5. Questionnaire

In general, the acceptance of self-sampling devices was high (Table 5). As a favourite device, 31.8% (21/65) of women preferred the Evalyn Brush and 27.7% (18/65) preferred the Colli-Pee, respectively. The FLOQSwab was preferred by 26.2% (17/65) of women. A total of 9.2% (6/65) of women were unsure about their favorite self-sampling device, making a total of over 95% (62/65) of women preferring self-sampling over a clinician-taken smear for their next cervical cancer screening. Only 4.6% (3/65) of women preferred the clinician-taken smear over one the self-samples.

When asked to choose between the two self-sampling smear devices Evalyn Brush and the FLOQSwab, 60% of patients preferred the Evalyn Brush and 38.5% preferred the FLOQSwab. One woman (1.5%) gave no answer. When asked to choose between the vaginal self-sampling or urine collection, 44.6% stated to prefer a vaginal self-sampling device over a urine-collection device. A total of 50.8% preferred the urine-sample device over one of the vaginal smear devices and 4.6% did not state a single preference

## 4. Discussion

In our open prospective multicenter trial, we evaluated the clinical performance, acceptability and feasibility of three different HPV self-testing devices. Clinician-taken smears, Evalyn Brush self-samples, FLOQSwab self-samples and the Colli-Pee self-samples all showed similar high sensitivity detecting CIN 3+ lesions in this study population. None of the devices missed a single cervical cancer case. High acceptance of self-sampling devices was shown as user-friendliness and ease of use was rated highly. Over 95% of the enrolled women preferred self-sampling over a clinician-taken smear for their next cervical cancer screening.

The high HPV detection rate of the self-sampling devices is consistent with a meta-analysis by Arbyn et al. (2014) [11]. This analysis supports the theory that self-samples could be used preventively to detected precancerous lesions at home for non-participants of screening. In this study population, more than 95% of women preferred one of the self-sample devices over the clinician-taken smear for their next cancer screening. These results, and the generally positive rating of the self-samples regarding ease of use, imply an overwhelming acceptance of these devices. Self-sampling could be a way to reach more women with precancerous lesions, since a screening uptake was found to be significant for self-sampling participants whether mailed directly to home or offered door-to-door [20].

In line with previous studies, the vaginal self-sampling device Evalyn Brush showed excellent sensitivity for the detection of CIN 2+ at 89.7% [17]. In addition to the established self-sampling device Evalyn Brush this study showed acceptable results for a dry vaginal self-sampling device (FLOQSwab), 78.5% and a self-sampling urine device (Colli-Pee) 73.8%, respectively. Especially for CIN 2+ lesions the first-void urine samples showed similar good results when compared to a vaginal self-sampling device, as it achieved a sensitivity of 77.6%, only about 5% less than the sensitivity of the FLOQSwab 82.7% [16]. These results agree with evidence of the meta-analysis by Pathak et al. (2014) [21] stating that first-void urine has a pooled sensitivity for high-risk HPV types of 77% (68% to 84%).

Overall, the Evalyn Brush performed best among self-sampling devices, in terms of detection of high-grade CIN. The agreement between the Colli-Pee and the other self-sampling devices was moderate (***K*** 0.57) for the Evalyn Brush and substantial (***K*** 0.75) for the FLOQSwab, respectively, showing no major inferiority of first-void urine to vaginal self-sampling in congruence with results of Cho et al. (2019) [22].

The questionnaire established the first-void urine-sampling device (Colli-Pee) as the most user-friendly of the three self-sampling devices since it ranked first among all specimen types in both categories and was, overall, well-accepted by the enrolled women, similar to results of Ketelaars et al. (2017) [23]. The Colli-Pee also ranked first in certainty of having taken a good sample but only third in comprehensibility of instruction. Overall, these results suggest that the Colli-Pee is a convenient screening tool for women at home, since the use is very simple and reliable for detecting CIN 2+ lesions. Possibly, urine sampling was preferred as it was considered less painful and more comfortable than having to insert a vaginal swab. Furthermore, having to take a “blind” vaginal swab seems to be not as assuring as taking a urine sample to the participants.

Both vaginal self-sampling devices scored similar results in all categories. Enrolled women rated the Evalyn Brush slightly better than the FLOQSwab.

Our study’s limitation is the cohort type, since we included only patients referred to a colposcopy clinic due to an abnormal PAP smear. Therefore a selection bias cannot be excluded. In this selected population, the CIN 2+ rate is 89.2%. In a screening population with a much lower incidence of CIN 2+, the results may vary. Therefore, our results cannot be extrapolated to the female population in general. Nevertheless, women not participating in a regular screening program, who are at a higher risk of developing invasive cervical cancer, could be motivated to participate by self-sampling devices instead.

The strength of this study is that every enrolled woman performed all three self-sampling at home with no medical assistance, simulating a realistic representation on how self-sampling devices could be used in a screening setting by mailing the device and an instructions for use directly to the patient. Our study also measured the acceptance and feasibility of the three self-sample devices and overall attitude towards self-sampling.

## 5. Conclusions

Our study shows the overall sensitivity, acceptability and feasibility of HPV-self sampling devices. Self-sampling devices show a high CIN 3+ detection rate comparable to that of a clinician-taken smear. The highly rated user-friendliness and simplicity of these devices was shown great acceptance by enrolled women. The Evalyn Brush had the highest hr-HPV and CIN 2+ sensitivity among self-sampling devices and was rated as the favorite method for cervical cancer screening. Nevertheless, FLOQSwab and Colli-Pee showed comparable results in hr-HPV sensitivity and especially in CIN 2+ detection rate. Self-collected first-void urine could be an alternative to dry vaginal self-sampling. The superior acceptance of urine sampling shows that this could be a promising option for women especially in regions where vaginal sampling might not be tolerated due to cultural barriers. Randomized, prospective, population-based trials are pending to establish the role of self-sampling devices for non-participators in the regular screening program in Germany.

## Figures and Tables

**Table 1:** HPV positivity according to histology in clinician-taken smears (CTS), self-collected urine samples (Colli-Pee), FLOQSwab and Evalyn Brush samples.

Histological Diagnosis	N	CTS	Colli-Pee	FLOQ Swab	Evalyn Brush
No dysplasia	2	1 (50%)	1 (50%)	1 (50%)	1 (50%)
CIN 1	5	3 (60%)	2 (40%)	1 (20%)	3 (60%)
CIN 2	28	24 (85.7%)	17 (60.7%)	21 (75%)	23 (82.1%)
CIN 3, AIS	25	23 (92%)	23 (92%)	23 (92%)	24 (96%)
Cx-Cancer	5	5 (100%)	5 (100%)	5 (100%)	5 (100%)
Total	65	56 (86.2%)	48 (73.8%)	51 (78.5%)	56 (86.2%)

**Table 2:** Sensitivity and specificity for CIN 2+ in clinician-taken smears (CTS), self-collected urine samples (Colli-Pee), FLOQSwab and Evalyn Brush samples (*n* = 65).

Type of Sample	TP	FN	FP	TN	Total	CIN 2+ Sensitivity [95% CI]	CIN 2+ Specificity [95% CI]
Clinician-taken smear	52	6	4	3	65	89.7% [81.7–97.6%]	42.9% [3.3–82.5%]
Colli-Pee	45	13	3	4	65	77.6% [66.8–88.4%]	57.1% [17.5–96.7%]
FLOQSwab	49	9	2	5	65	82.8% [73.0–92.6%]	71.4% [35.3–100%]
Evalyn Brush	52	6	4	3	65	89.7% [81.7–97.6%]	42.9% [3.3–82.5%]

**Table 3:** Relative sensitivity and specificity for CIN 2+ (*n* = 65).

Comparison	Relative Sensitivity [95% CI]	Relative Specificity [95% CI]
Colli-Pee versus CTS	0.87 [0.68–1.08]	1.33 [0.21–29.3]
FLOQSwab versus CTS	0.92 [0.75–1.13]	1.66 [0.43–30.3]
Evalyn Brush versus CTS	1.00 [0.84–1.19]	1.00 [0.04–25.0]
Colli-Pee versus FLOQSwab	0.94 [0.72–1.21]	0.80 [0.18–2.74]
Colli-Pee versus Evalyn Brush	0.87 [0.68–1.08]	1.33 [0.21–29.3]
FLOQSwab versus Evalyn Brush	0.92 [0.75–1.13]	1.66 [0.43–30.3]

**Table 4:** Agreement on HPV positivity in clinician-taken smear (CTS), self-collected urine samples (Colli-Pee), FLOQSwab and Evalyn Brush.

Comparison of Samples (*n* = 65)	Concordant	Discordant	Cohen’s Kappa K
CTS versus Colli-Pee	51	14	0.34
CTS versus FLOQSwab	51	14	0.29
CTS versus Evalyn Brush	57	8	0.48
Colli-Pee versus FLOQSwab	59	6	0.75
Colli-Pee versus Evalyn Brush	56	9	0.57
FLOQSwab versus Evalyn Brush	58	7	0.63

**Table 5:** Acceptance of self-sampling devices according to difficulty of use, comprehensibility of the instructions, certainty of generating a valid sample and comfortability of use ranging from 1 (worst possible score) to 10 (best possible score).

Type of Sample	Difficulty of Use [95% CI]	Comprehensibility of Instruction [95% CI]	Certainty of Generating a Valid Sample [95% CI]	Comfortability of Use [95% CI]
Colli-Pee	8.1 [7.7–8.5]	8.1 [7.6–8.5]	7.7 [7.2–8.3]	8.1 [7.7–8.5]
FLOQSwab	7.9 [7.5–8.3]	8.2 [7.9–8.6]	6.3 [5.6–6.9]	7.1 [6.5–7.7]
Evalyn Brush	8.0 [7.6–8.3]	8.3 [7.9–8.6]	6.8 [6.2–7.4]	7.3 [6.8–7.8]
Clinician-taken smear	n.a.	n.a.	n.a.	5.5 [4.4–6.7]

## Data Availability

Data from this study will be provided upon request.

## Supplementary Materials

The following are available online at https://www.mdpi.com/article/10.3390/ijerph181910388/s1, Three representative pictures of PCR amplification curves (Evalyn, FLOQSwabs and Colli-Pee).
