# Supplementary figures and images for: CoCoss-Trial: Concurrent Comparison of Self-Sampling Devices for HPV-Detection
Source: Int J Environ Res Public Health. 2021 Oct 2;18(19):10388. doi: 10.3390/ijerph181910388 (PMC8508434; doi:10.3390/ijerph181910388)

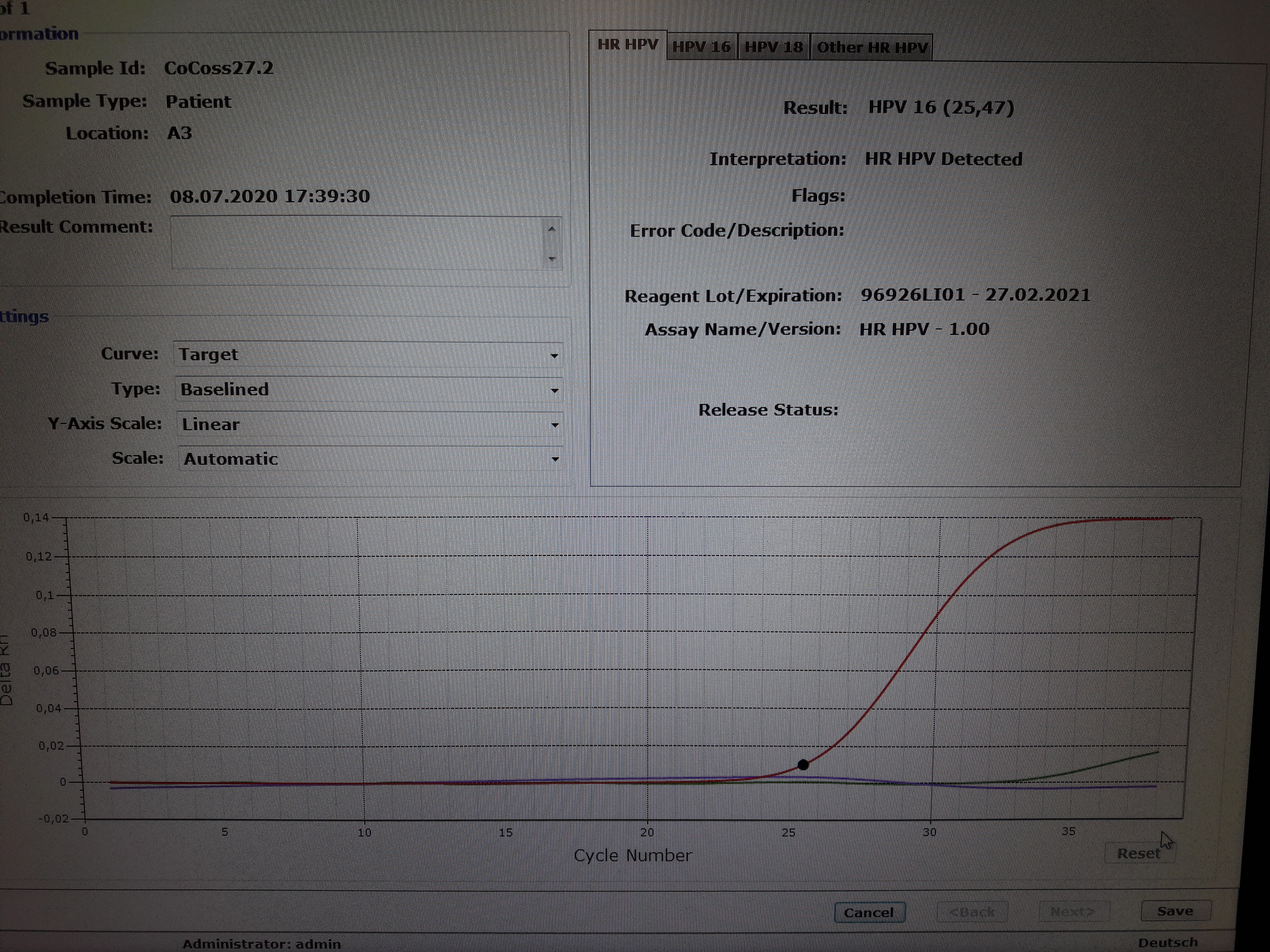

Supplement: Supplementary file 1 [file ijerph-18-10388-s001.zip › Amplicifation urine.jpg]

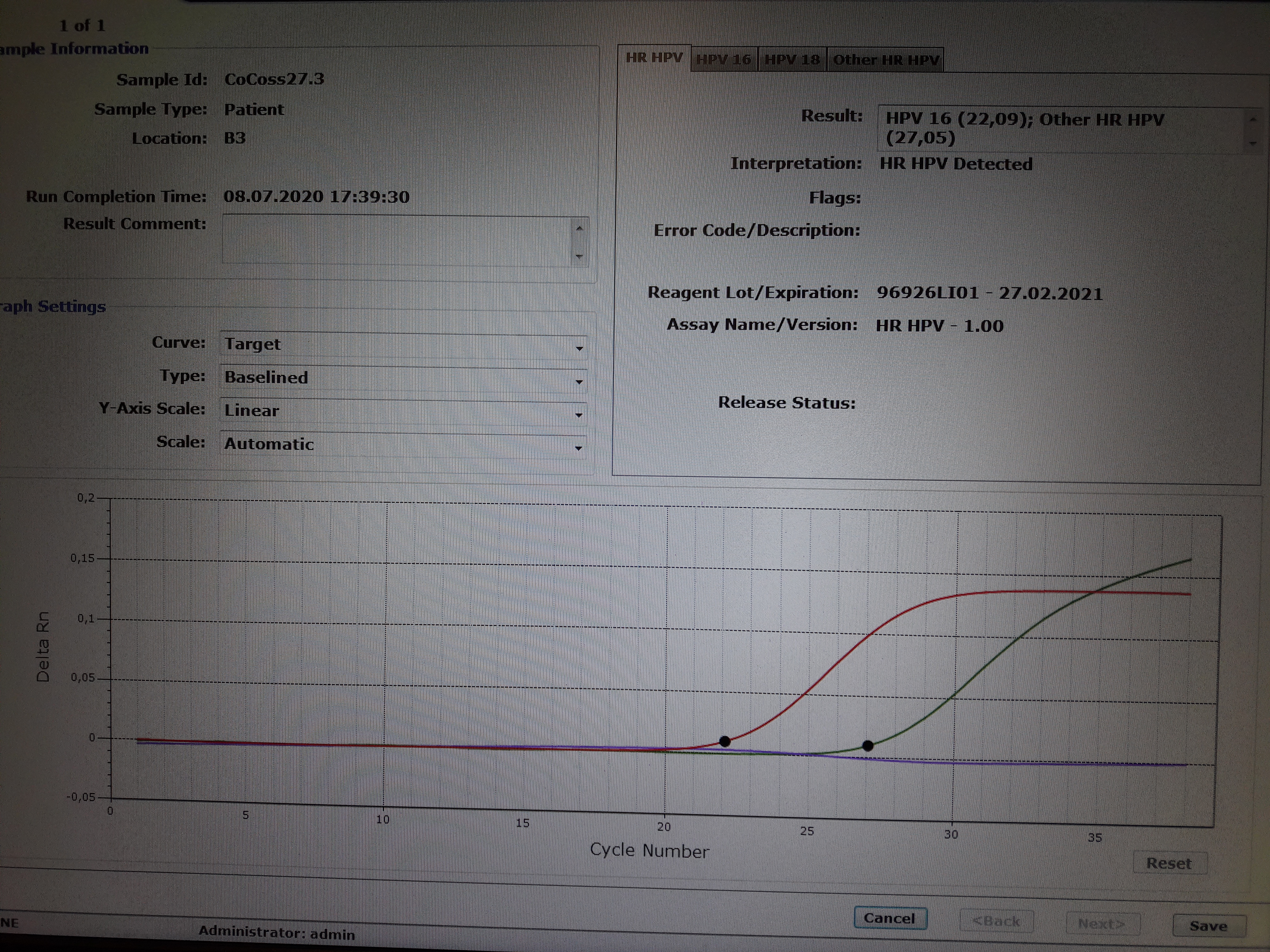

Supplement: Supplementary file 1 [file ijerph-18-10388-s001.zip › Amplification Evalyn.jpg]

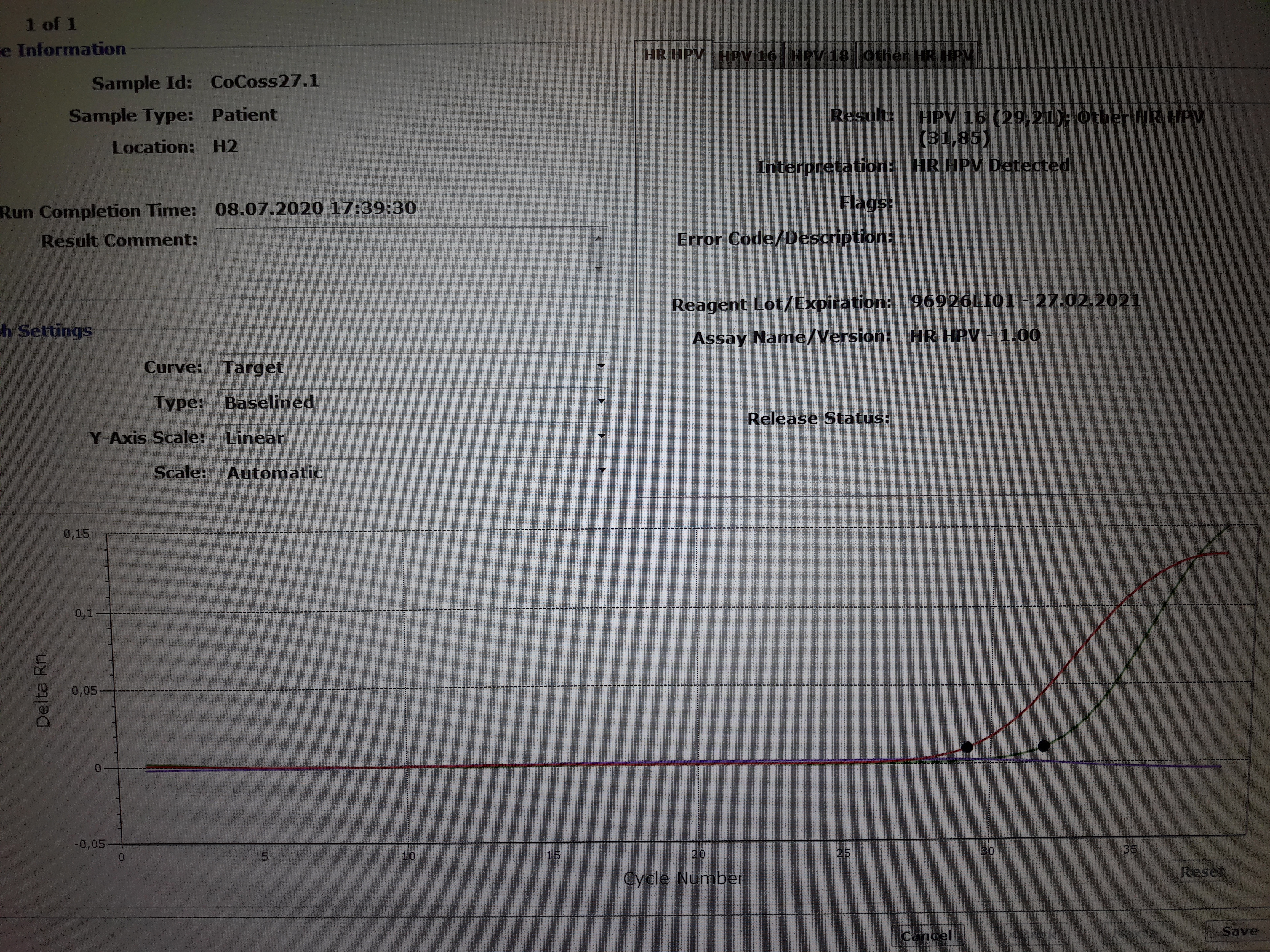

Supplement: Supplementary file 1 [file ijerph-18-10388-s001.zip › Amplification FloqSwabs.jpg]
